# Extracellular Vesicle Associated Proteomic Biomarkers in Breast Cancer: A Systematic Review and Meta-Analysis

**DOI:** 10.3390/cells15030231

**Published:** 2026-01-26

**Authors:** Nahad Al-Mahrouqi, Hasan Al-Sayegh, Shoaib Al-Zadjali, Aafaque Ahmad Khan

**Affiliations:** Research Laboratories, Sultan Qaboos Comprehensive Cancer Care & Research Center, University Medical City, Al Khoud, Muscat 123, Oman

**Keywords:** breast cancer, extracellular vesicles, proteomics, protein biomarkers, pathway enrichment, tumor progression

## Abstract

Breast cancer continues to be the most frequently diagnosed cancer among women worldwide and remains a leading cause of cancer-related mortality. Despite advances in imaging and biopsy-based approaches, current diagnostic methods are invasive, costly, and often insufficient to capture the molecular heterogeneity of tumors. Extracellular vesicles (EVs) have emerged as promising non-invasive biomarkers owing to their role in intercellular communication and their enrichment with tumor-specific cargo. This study conducted a systematic review and meta-analysis of published literature to investigate proteomic alterations in EVs derived from breast cancer samples. From an initial 1097 records screened, four eligible studies were identified, reporting 628 differentially expressed proteins, of which 38 were consistently observed across multiple datasets. Functional enrichment analyses revealed predominant localization of these proteins to vesicle-associated compartments and significant involvement in biological processes related to cell growth, immune regulation, and tumor progression. Pathway analysis further highlighted integrin-mediated interactions, platelet activation, and hemostasis pathways as key molecular mechanisms represented within breast cancer EVs. Overall, the findings reveal a distinct EV proteomic signature in breast cancer that could support early detection and patient monitoring through minimally invasive testing. Future large-scale and standardized studies are needed to validate these candidate proteins and advance EV proteomics toward clinical application in breast cancer management.

## 1. Introduction

In 2020, approximately 2.3 million new cases of breast cancer were diagnosed in women worldwide, making it the most commonly occurring cancer among women and a leading cause of cancer-related deaths [1]. Despite remarkable advances in screening and treatment, breast cancer continues to impose a major public health burden globally. Early detection remains crucial, as timely diagnosis significantly reduces mortality and improves therapeutic outcomes [2]. Current diagnostic and monitoring strategies for breast cancer rely primarily on imaging techniques such as mammography, ultrasound, PET/CT, and MRI, often followed by tissue biopsy for histopathological confirmation and molecular profiling [3,4].

Although imaging and biopsy remain the mainstays of diagnosis, they are costly, invasive, and less accurate for certain breast cancer subtypes especially in low-resource settings [5]. Furthermore, breast tumors exhibit marked intra-tumoral and intertumoral heterogeneity, which complicates accurate diagnosis and the selection of personalized treatment strategies [6]. Tissue biopsy, while regarded as the gold standard, provides only a static snapshot of the tumor and often fails to capture spatial and temporal genomic heterogeneity. It is also unsuitable for serial monitoring of disease progression or therapeutic response, thereby restricting its routine clinical utility [7]. These limitations have led to growing interest in non-invasive diagnostic tools that can complement or replace conventional biopsy approaches. Among such emerging technologies, *liquid biopsy* has gained prominence for its ability to analyze tumor-derived materials circulating in body fluids, thereby providing a dynamic representation of tumor biology. Liquid biopsy involves the assessment of circulating tumor cells (CTCs), cell-free nucleic acids, proteins, and extracellular vesicles (EVs), offering a minimally invasive means for early detection, prognostication, and real-time monitoring of disease [8]. This approach holds promise for identifying molecular signatures that can guide therapeutic decisions and enable longitudinal tracking of tumor evolution.

Extracellular vesicles (EVs), a heterogeneous population of lipid bilayer-enclosed particles, including microvesicles and exosomes, have emerged as a key component of liquid biopsy. EVs are actively secreted by nearly all cell types and carry a cargo of proteins, nucleic acids, lipids, and metabolites that reflect the physiological or pathological state of their cell of origin [9]. In cancer, tumor-derived EVs are enriched with bioactive molecules that participate in intercellular communication, metastasis, and immune modulation. They also offer an attractive alternative to traditional biomarkers, as their membrane encapsulation protects cargo from enzymatic degradation, enhancing their stability in circulation [10]. Numerous studies have reported that EV-associated proteins exhibit cancer type–specific expression patterns and can serve as potential biomarkers for early detection, disease monitoring, and therapeutic response [11]. Importantly, variations in the composition and abundance of EV proteins can reveal not only tumor classification and stage but also biological processes underlying cancer progression [12].

Given these promising attributes, proteomic profiling of extracellular vesicles has become an important and fast-growing area in cancer research. However, pre-analytical variables are known to strongly influence the accuracy of EV proteomic measurements, particularly when EVs are isolated from blood-based biofluids. Plasma- and serum-derived preparations often contain substantial amounts of co-isolated lipoproteins, especially LDL and VLDL particles, which closely mimic EV size and density and thereby introduce non-vesicular proteins into EV proteomic datasets [13]. Differences in isolation speed and centrifugal force can further alter EV yield and selectively enrich subpopulations with distinct proteomic signatures, leading to inconsistent protein identification across studies [14]. High-speed centrifugation and precipitation-based methods have also been shown to induce EV aggregation or co-purify protein complexes, thereby distorting the true vesicle-associated proteome [15]. In addition, EVs can acquire a protein corona from surrounding biofluids, altering their surface composition and introducing artifacts in mass-spectrometry–based detection [16]. This systematic review and meta-analysis were therefore designed to fill this gap by critically examining published studies that analyzed differentially expressed proteins in EVs derived from breast cancer patients in comparison with healthy controls.

The main objectives of this study were to first, compile and summarize proteins that were consistently reported as dysregulated across multiple studies; second, assess overall expression patterns using meta-analysis and vote-counting methods; and third, interpret the biological significance of these proteins through functional enrichment and pathway analyses. By integrating data from available literature, this study aims to provide a clear and comprehensive overview of EV-associated proteomic alterations in breast cancer and to highlight promising biomarker candidates for future clinical validation and application.

## 2. Materials and Methods

### 2.1. Study Design

This systematic review was conducted to compile and evaluate published research focused on proteomic biomarkers in breast cancer extracellular vesicles. The review process adhered to the Preferred Reporting Items for Systematic Reviews and Meta-Analyses (PRISMA) guidelines [17] to maintain methodological transparency, accuracy, and reproducibility. This review protocol was not registered in PROSPERO due to the rapid timeline of the project and because the scope of the study, which involved synthesizing previously published proteomic datasets, did not involve patient-level intervention or outcomes requiring prospective preregistration.

### 2.2. Search Strategy and Selection Criteria

A comprehensive search was carried out in March 2025 across four electronic databases: National Center for Biotechnology Information (NCBI), Medline, Academic Search Ultimate, and CINAHL Plus. The search terms included “extracellular vesicles” OR “microvesicles” OR “exosome” AND “breast cancer” AND “proteomics”. The complete database-specific search strings are mentioned in the Supplementary Methods Section S1.

The search covered all available publication years, as no date restrictions were applied. The final search was conducted on 1 October 2025. Only English-language, peer-reviewed original research articles were included. Reviews, conference abstracts, commentaries, non-English publications, and studies without accessible full text were excluded.

All identified studies were imported into Zotero for reference management and removal of duplicates [18]. In Zotero, duplicates were identified and removed using automated matching based on DOI, title, and author names, followed by manual verification. Two reviewers independently screened titles, abstracts, and full texts. Any discrepancies were resolved through discussion and consensus. Studies were included if they met the following criteria: (1) examined proteomic biomarkers within extracellular vesicles, microvesicles, or exosomes in breast cancer; (2) reported differential protein data between cancer and normal samples; and (3) involved human participants or cell lines. Studies were excluded if they were reviews, conference abstracts, or did not meet the inclusion criteria. Included studies comprised discovery-phase proteomic analyses of EVs isolated from plasma, serum, or cultured breast cancer cell lines. A summary of key methodological features of all included studies—including sample source, EV isolation approach, characterization markers, and MS workflow—is provided in Supplementary Table S1.

### 2.3. Data Extraction

Titles and abstracts were screened to select eligible studies. Two reviewers (NM and AAK) independently extracted data from the eligible studies using a structured data sheet, which included information on authors, publication year, sample size, sample type, fold changes and *p*-values of proteomic biomarkers, and the proteomics approach applied.

### 2.4. Meta-Analysis

Meta-analysis and vote counting were carried out using the Amanida R-package (version 0.2.3) [19]. This package combines *p*-values and fold-change values from individual studies, applying weights according to sample size so that larger studies contribute more. The Fisher test was used to calculate combined *p*-values, while fold-change values were log-transformed and averaged using weighted calculations based on study size. The Amanida framework combines significance and effect-size information using Fisher’s method and weighted median fold changes; it does not generate conventional heterogeneity statistics (e.g., I^2^, τ^2^, or influence diagnostics). Given the small number of included studies and the methodological diversity across EV isolation workflows and sample matrices, formal heterogeneity estimation was not feasible. As such, the meta-analytic findings should be interpreted as a descriptive integration of available proteomic evidence rather than as a variance-based random-effects model.

### 2.5. Functional Enrichment and Pathway Analysis

A list of significantly differentially expressed proteins was generated from the dataset based on the following criteria: (1) expression levels in cancer samples at least two-fold higher than in control samples, and (2) a *p*-value of 0.05 or lower to confirm statistical significance. Functional enrichment and pathway analyses of the selected proteins were carried out using the FunRich tool (version 3.1.4) [20] to explore their biological roles and associated pathways. Enrichment analyses were performed using the FunRich default EV background, as most included studies did not provide global protein lists, making a unified custom background unfeasible. Functional associations among proteins identified by mass spectrometry analysis were examined using the STRING database ((version 9.1, [http://string-db.org/]) [21], accessed on 15 September 2025). STRING provides an extensive resource of protein–protein interactions by integrating experimental data, predicted associations, and information obtained through automated text mining. For this analysis, the minimum required interaction score was set to high confidence (0.7), with the number of interactors limited to 10. We used FDR (Benjamini–Hochberg) correction for all enrichment analyses, considering terms significant at q < 0.05. Amanida meta-analytic *p*-values are unadjusted; proteins were retained when combined *p* < 0.05 and fold-change direction was consistent. Fold-change significance thresholds followed each study’s reported criteria.

## 3. Results

**Figure 1:**
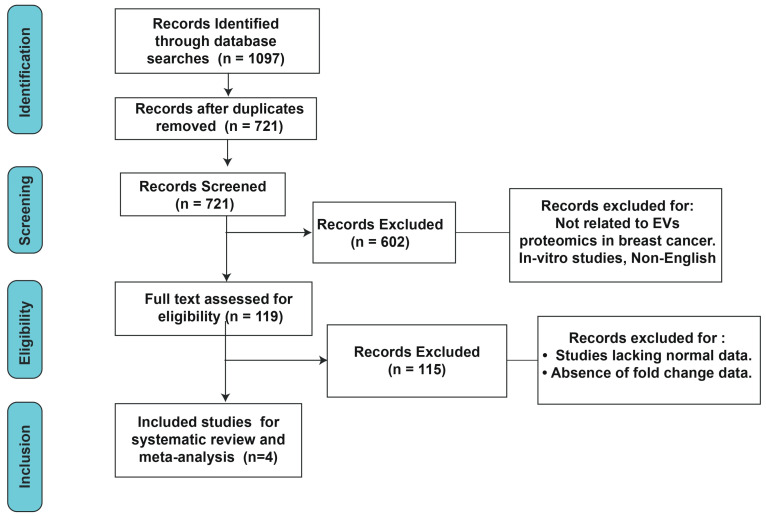
Flowchart depicting the steps of study selection.

The systematic literature search initially identified a total of 1097 records across the selected databases, as outlined in Figure 1. After removal of duplicate entries, 721 unique records remained. These records were then screened on the basis of titles and abstracts to assess their relevance. During this process, 602 studies were excluded because they did not address extracellular vesicle proteomics in breast cancer. The remaining 119 articles were subjected to full-text evaluation to determine their eligibility. Following this detailed review, 115 articles were excluded for reasons such as not focusing specifically on EV-derived proteins in breast cancer or not providing data on the differential expression (fold change) of proteins in EVs between breast cancer patients and healthy controls. Ultimately, 4 studies fulfilled all inclusion criteria and were selected for comprehensive analysis.

**Figure 2:**
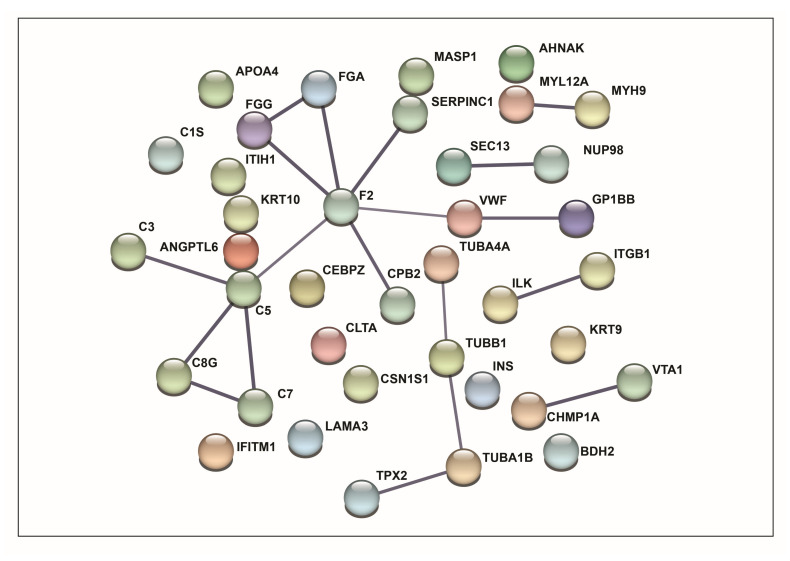
STRING network of the 38 proteins identified across multiple studies, showing predicted protein–protein interactions. Nodes represent proteins and edges indicate functional associations based on STRING confidence scores.

From each of these included studies, detailed information was extracted, including the name and symbol of differentially expressed proteins, fold change values, associated *p*-values, cohort characteristics (such as sample type and size), and the mass spectrometry (MS) platform employed (Supplementary Table S2). Altogether, 628 proteins were identified as differentially expressed in extracellular vesicles derived from breast cancer patients compared with healthy individuals (Supplementary Table S2). Among these, 38 proteins were consistently reported across more than one study, indicating stronger evidence of their potential role as biomarkers, as presented in Supplementary Table S3. For these commonly identified proteins, protein–protein interaction analysis was performed using the STRING network to identify functionally interconnected proteins (Figure 2). The analysis included differentially expressed proteins along with 10 additional interactors. The resulting network was visualized in confidence view, where proteins were represented as nodes and their associations as connecting edges. Edge thickness corresponded to interaction strength, with thicker lines indicating stronger associations and thinner lines representing weaker ones.

### 3.1. Functional Enrichment Analysis

**Figure 3:**
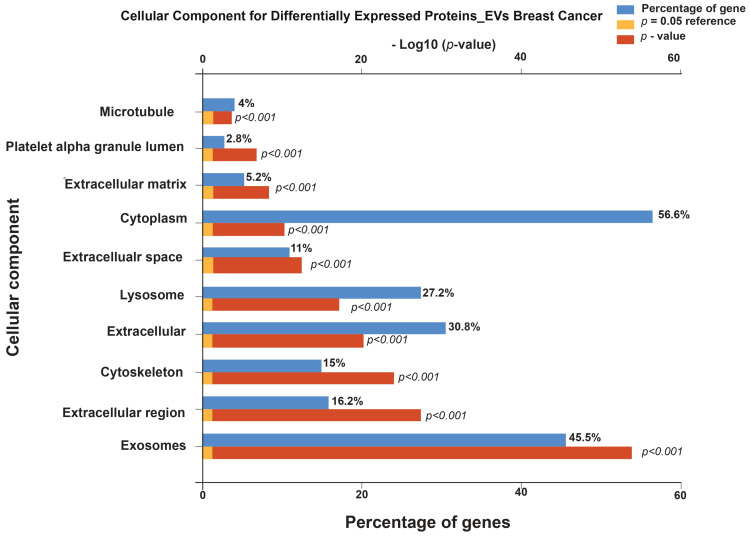
Cellular component enrichment analysis of differentially expressed proteins in extracellular vesicles (EVs) from breast cancer patients. The bar plot shows the percentage of genes associated with each cellular compartment (blue bars), alongside the statistical significance of enrichment (−log_10_ *p*-value, red bars).

A gene ontology and pathway enrichment analysis of significantly expressed proteins was carried out using FunRich. Cellular component enrichment analysis of significantly expressed proteins in extracellular vesicles (EVs) from breast cancer patients showed strong localization to EV-related compartments. The most enriched components included the cytoplasm (56.6%), exosomes (45.5%), extracellular (30.8%), extracellular region (16.2%), cytoskeleton (15%), and extracellular space (11%), all with highly significant values (*p* < 0.001) (Figure 3). Additional enrichment was observed in lysosomes (27.2%), extracellular matrix (5.2%), microtubules (4%), and platelet alpha granule lumen (2.8%), each also reaching statistical significance (*p* < 0.001).

Gene ontology analysis of EV proteins highlighted several biological processes (Supplementary Figure S1). Cell growth and/or maintenance (17%) and immune response (7.7%) showed highly significant enrichment (*p* < 0.001), while protein metabolism was also significantly enriched (12.6%, *p* = 0.007). Although, signal transduction (26%) and cell communication (24.7%) were the most represented categories based on gene percentage; however, both were not statistically significant (*p* = 1). Other processes, such as spindle assembly, blood vessel development, immune cell migration, coagulation regulation, and development, had very low gene representation (≤0.4%) and did not reach statistical significance (*p* = 1).

### 3.2. Pathway Analysis

For pathway analysis, gene identifiers of significantly expressed proteins were imported into FunRich, and the top 10 enriched pathways for each cancer type were identified. The pathway enrichment analysis of proteins revealed significant involvement in pathways associated with cell adhesion, signaling, and tumor progression. The top 10 enriched pathways, along with their *p*-values, are represented in Table 1. Among these, Integrin family cell surface interactions showed the highest level of significance (*p* = 1.42 × 10^−8^), with 96 proteins mapped to this pathway, including ITGA6, ITGB1, FN1, ACTN1, TLN1, and PXN. Closely related pathways, such as Beta1 integrin cell surface interactions and alpha9 beta1 integrin signaling events, were also enriched. Additional pathways significantly represented included Proteoglycan syndecan-mediated signaling, Syndecan-1 signaling, and the Glypican pathway. TRAIL signaling and PAR1-mediated thrombin signaling were further enriched, with key proteins such as GSK3B, STAT5A/B, CTNNB1, and NFKB2 commonly appearing across multiple pathways.

**Figure 4:**
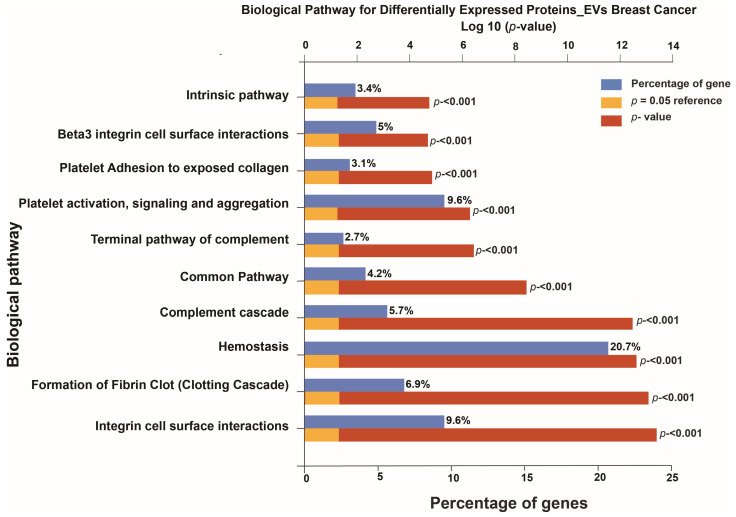
Enriched biological pathways of differentially expressed extracellular vesicle proteins in breast cancer. Bar chart displays the top enriched pathways based on gene percentage (blue bars) and significance levels (red bars), with reference line at *p* = 0.05 (orange).

The Hemostasis pathway was the most enriched overall, with 20.7% of EV proteins involved (*p* < 0.001). Integrin cell surface interactions and Platelet activation, signaling, and aggregation followed, each accounting for 9.6% of proteins. Other enriched pathways included formation of fibrin clot (6.9%), complement cascade (5.7%), and Beta3 integrin cell surface interactions (5%). Additional pathways such as the intrinsic pathway, platelet adhesion to exposed collagen, and the terminal complement pathway were also significantly represented, although with lower percentages of proteins. All identified pathways showed high statistical significance (*p* < 0.001). Figure 4 depict the top 10 altered biological pathways among differentially expressed proteins in EVs of breast cancer patients.

### 3.3. Meta-Analysis

**Figure 5:**
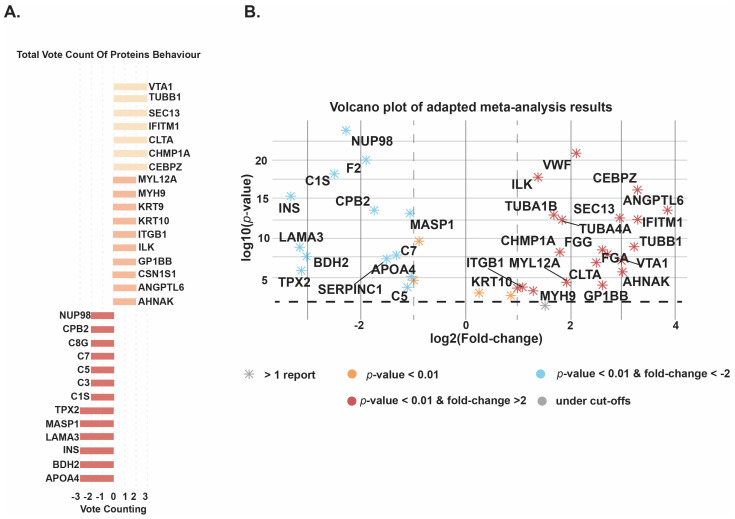
(**A**) Vote counting analysis of EV-associated protein expression in breast cancer compared to healthy controls. (**B**) Volcano plot showing log_2_ fold change versus −log_10_ *p*-value for EV proteins in breast cancer patients compared to healthy controls. Significantly upregulated proteins (red, log_2_FC > 2, *p* < 0.05). Downregulated proteins (blue, log_2_FC < −2, *p* < 0.05). Orange points represent proteins with statistically significant but moderate fold changes (|log_2_FC| < 1), and gray points indicate non-significant differences.

For the meta-analysis, we applied strict inclusion criteria and considered only those proteins that were significantly expressed and supported by at least two independent studies. This approach minimized the risk of bias from single-study findings and ensured that the results reflected consistent trends across different datasets. For each protein that met these criteria, a combined *p*-value and fold change were calculated by integrating data from the individual studies. In addition, a vote counting method was used to highlight proteins that were repeatedly reported as upregulated or downregulated in extracellular vesicles (EVs) derived from breast cancer samples compared with controls. Vote count analyses illustrating the significantly expressed proteins are presented in Figure 5A. The analysis identified a group of proteins that showed consistent upregulation across multiple studies. Notably, VTA1, TUBB1, SEC13, IFITM1, CLTA, CHMP1A, and CEBPZ were reported as upregulated in three separate studies, strengthening the evidence for their association with breast cancer–derived EVs. Another set of proteins, including MYL12A, MYH9, KRT9, KRT10, ITGB1, CSN1S1, ANGPTL6, and AHNAK, were found to be upregulated in two different studies, further supporting their potential role as biomarkers. On the other hand, several proteins were consistently found to be downregulated in breast cancer EVs. APOA4, BDH2, INS, LAMA3, MASP1, and TPX2 were each reported as downregulated in three separate analyses, while proteins such as C1S, C3, C5, C7, G8G, CPB2, and NUP98 were reported as downregulated in two studies (vote count = −2).

Analysis of extracellular vesicle (EV) proteins from breast cancer patients compared with healthy controls revealed a distinct pattern of differential expression, as shown in the volcano plot (Figure 5B). A total of 23 proteins were found to be significantly upregulated (log_2_ fold change > 1, *p* < 0.05). Among these, angiopoietin-like protein 6 (ANGPTL6) displayed the highest increase in expression (fold change = 14.6, *p* = 2.69 × 10^−14^). Other strongly upregulated proteins included IFITM1 (fold change = 9.97), CEBPZ (9.7), TUBB1 (9.3), AHNAK (8.0), and VTA1 (7.9), all of which were consistently enriched in cancer–derived EVs. In contrast, 15 proteins showed significant downregulation (log_2_ fold change < −2, *p* < 0.05). These included INS (fold change = 0.10), LAMA3 (0.11), TPX2 (0.11), BDH2 (0.12), C1S (0.18), NUP98 (0.21), and F2 (0.27).

## 4. Discussion

This systematic review brings together current evidence on extracellular vesicle (EV)–associated proteins that show altered expression in breast cancer. Our analysis indicates that breast cancer–derived EVs contain a distinct proteomic profile, with several proteins consistently reported across independent studies. These findings strengthen the view that EVs are active participants in tumor biology rather than passive by-products of cellular turnover, carrying molecular cargo that reflects the state and behavior of their cells of origin.

Extracellular vesicles are membrane-enclosed structures that mediate intercellular communication through the transfer of proteins, lipids, and nucleic acids [22,23]. The cellular component enrichment observed in this review confirmed that the identified proteins predominantly localized to EV-associated compartments, supporting the quality of isolation methods used in the included studies. A large fraction of these proteins originated from the cytoplasm and endosomal compartments, consistent with the regulated biogenesis of exosomes through multivesicular bodies [24]. These vesicles are known to be released via both ESCRT-dependent and ESCRT-independent pathways, which control selective cargo loading [25,26]. Cvjetkovic et al. (2016) [27] previously reported that more than half of the surface-accessible EV proteins were of cytosolic origin, a finding that aligns with the distribution patterns observed in our dataset. Together, these observations indicate that the proteomic profiles compiled here reliably capture the molecular composition of breast cancer–derived EVs and form a valid basis for biological interpretation.

The gene ontology and pathway enrichment analyses revealed that many significantly expressed proteins in breast cancer–derived EVs were involved in biological processes central to tumor progression, including signal transduction, intercellular communication, and immune modulation. These findings are consistent with the established role of EVs as molecular messengers that transport diverse biomolecules—such as proteins, nucleic acids, and lipids—to influence recipient cell behavior [28]. The prominence of signaling and communication pathways reflects the function of EVs as key mediators of intercellular exchange within the tumor microenvironment [12]. Although enrichment in these pathways did not reach statistical significance (*p* = 1), this pattern likely represents a fundamental feature of EV proteomes rather than a breast-cancer-specific signature, as such processes are intrinsic to EV-mediated signaling across both normal and diseased tissues. By contrast, processes associated with cell growth, maintenance, and immune response showed significant enrichment, suggesting a more direct link to the tumor phenotype. Tumor-derived EVs have been shown to carry immunomodulatory and tumor antigens that can suppress immune surveillance or alter immune-cell activity [29]. Supporting this, Santoro et al. (2023) demonstrated that EVs isolated from luminal B (BT474) and triple-negative (HS578T) breast cancer cell lines modulated the activity of natural killer cells and regulatory T cells, indicating that EV cargo can reshape immune dynamics within the tumor microenvironment [30]. Similar observations were made by Ozawa et al. (2018) and Leone et al. (2024) [31,32], who demonstrated that extracellular vesicles released from aggressive breast cancer cell lines can modulate the behavior of non-malignant cells. Ozawa et al. showed that EVs derived from triple-negative *HCC1806* cells enhanced proliferation and drug resistance in non-tumorigenic *MCF10A* cells by activating pro-survival signaling pathways [31]. Likewise, Leone et al. reported that EVs from *MDA-MB-231* and *HS578T* cells increased proliferation, migration, and invasiveness in normal *BEAS-2B* epithelial cells, accompanied by features of epithelial–mesenchymal transition [32]. Beyond their structural and adhesive roles, breast cancer–derived extracellular vesicles (EVs) can actively modulate oncogenic signaling in recipient cells. Integrin-dependent EV uptake has been shown to facilitate downstream activation of kinase pathways, providing a mechanistic link between EV-associated integrins and pro-survival signaling networks. Notably, ITGB3-mediated internalization of small EVs enhances signaling competence in breast cancer cells [33], supporting activation of pathways that converge on PI3K/AKT signaling. In addition to surface-mediated effects, EV cargo can directly engage this pathway; for example, CCL18-stimulated metastatic breast cancer cells secrete exosomal miR-760 that promotes malignant phenotypes in less aggressive cells through an ARF6–Src–PI3K–Akt axis [34]. EV-mediated suppression of negative regulators such as PTEN, including through miR-221/222, can further sustain PI3K/AKT activity and downstream transcriptional programs linked to tumor growth and stem-like properties [35].

Breast cancer–derived EVs were found to be enriched with proteins involved in pathways central to cell adhesion, signaling, and tumor progression. Among these, integrin-mediated interactions emerged as a dominant pathway, followed by Beta1 integrin cell surface interactions. Integrins are evolutionarily conserved and ubiquitously expressed adhesion receptors that anchor cells to the extracellular matrix while simultaneously transmitting biochemical and mechanical signals. Through this dual function, integrins regulate key cellular processes, including growth, survival, proliferation, and migration [36]. The integrin family is composed of 18 α subunits and 8 β subunits, which combine in specific α–β pairings to form unique heterodimers, each with distinct cellular functions [37]. One notable example is α6-integrin (ITGA6), which plays a critical role in mediating adhesion between cells and the surrounding stromal environment, thereby contributing to proliferation, migration, differentiation, and survival [38]. ITGA6 can associate with either the β1 (ITGB1) or β4 (ITGB4) subunit, forming α6β1 or α6β4 heterodimers, respectively [39]. Under physiological conditions, ITGA6 is expressed at low or undetectable levels in most normal tissues; however, it is frequently upregulated in tumor cells, where it has been implicated in cancer progression [40,41]. Asada et al. (2022) further reported that the *ITGA6A* splice variant is not only highly expressed in pancreatic cancer cell lines but is also enriched in extracellular vesicles isolated from the blood of patients with pancreatic ductal adenocarcinoma (PDAC), highlighting its potential as a circulating biomarker [42].

The interpretation of pathway enrichments must be tempered by the small number of eligible studies and the heterogeneity of sample matrices (plasma, serum, urine, and cell-line conditioned medium). In particular, plasma and serum EV proteomes are known to be susceptible to contamination from platelet activation, coagulation proteins, and circulating lipoproteins, all of which can co-isolate with EVs and artificially inflate pathways related to hemostasis or platelet function. Prior work has shown that even minimal platelet activation can release large quantities of platelet-derived vesicles and granule proteins that overshadow true circulating EV signals [43,44]. Similarly, lipoprotein particles such as LDL, VLDL, and HDL co-purify with EVs under density ranges commonly used in ultracentrifugation, contributing apolipoproteins and complement factors that bias downstream proteomic readouts [13,45]. These issues may partly explain the strong hemostasis- and platelet-related pathway enrichments observed in the plasma-derived datasets of our review. To mitigate such confounding, EV methodological guidelines recommend the use of platelet-poor plasma (PPP) preparation, additional high-speed spins, and size-exclusion chromatography (SEC) or density gradient ultracentrifugation, each of which reduces contamination from platelets, lipoproteins, and abundant plasma proteins [46,47]. However, these controls were not uniformly applied across the included studies, and therefore the enrichment patterns reported here should be interpreted with caution. Future EV proteomics studies would benefit from standardized pre-analytical workflows and robust contamination controls to more confidently delineate disease-related EV signatures.

The proteomic findings from this review draw attention to several extracellular-vesicle (EV) proteins that are functionally relevant to breast cancer biology. Among these, integrin β1 (*ITGB1*), myosin heavy chain (*MYH9*), von Willebrand factor (VWF), angiopoietin-like 6 (ANGPTL6), laminin α3 (LAMA3), and the scaffold protein AHNAK have been consistently reported in breast cancer proteomic or EV-associated datasets [48,49,50]. These proteins participate in key processes such as adhesion, motility, cytoskeletal organization, angiogenesis, and epithelial–mesenchymal transition pathways central to invasion and metastatic spread. Their recurring identification in breast cancer EVs suggests that tumor cells selectively load such molecules into vesicles as part of a coordinated strategy to remodel the surrounding microenvironment, enhance vascular interactions, and communicate pro-tumorigenic signals to distant sites. In parallel, other frequently detected proteins, including vesicle-trafficking and structural components such as VTA1, CHMP1A, CLTA, and the tubulin isoforms TUBA1B and TUBB1, represent the machinery responsible for EV biogenesis and secretion [10,51]. Their enrichment in breast cancer EVs reflects an increased demand for vesicle production and turnover in malignant cells, which may facilitate the higher rate of molecular exchange typical of aggressive tumors. Together, these findings indicate that the EV proteome captures two complementary aspects of breast cancer biology: the presence of disease-linked effectors actively driving tumor progression and the upregulation of vesicular pathways that sustain intercellular communication and therapeutic resistance. This dual signature not only enhances our understanding of how breast cancer cells exploit vesicle networks but also points toward a focused group of EV-associated proteins with potential diagnostic and prognostic value deserving of further experimental validation. To aid interpretation across sample types, the matrix of origin for all EV proteins is now clearly indicated in Supplementary Table S2. Each matrix may introduce distinct biases—for example, lipoprotein carryover in plasma, protein dilution in urine, and culture-related artifacts in cell-line EVs—which should be considered when comparing results across studies.

To support clinical translation, these findings suggest a focused panel of EV-associated proteins that warrant orthogonal validation in independent patient cohorts. Proteins repeatedly identified across studies—such as ITGB1, ITGA6, ANGPTL6, VWF, MYH9, VTA1, CHMP1A, CLTA, and TUBB1—represent candidates for targeted verification using ELISA or mass spectrometry–based assays, including parallel reaction monitoring (PRM) or selected reaction monitoring (SRM). A prospective validation study would require pre-registered clinical endpoints (e.g., early diagnosis, subtype discrimination, or disease monitoring) and harmonized pre-analytical workflows, including standardized platelet-poor plasma preparation, uniform EV isolation procedures, and controlled storage conditions. Sample size calculations should be guided by expected effect sizes from discovery datasets, with at least 50–100 participants per clinical arm generally needed to achieve adequate power for biomarker validation. Such a structured validation framework would help clarify the clinical utility of EV-derived protein biomarkers and support eventual translation into diagnostic or monitoring assays.

Several of the proteins consistently identified across EV studies also have evidence of clinical relevance in breast cancer patients. High expression of ITGB1 in breast tumors has been correlated with worse overall and disease-free survival in meta-analyses of clinical cohorts, supporting its potential as a prognostic marker [52]. Similarly, elevated ITGA6 (integrin α6) expression has been documented in a substantial proportion of invasive breast carcinomas and is associated with reduced patient survival [52]. Elevated plasma levels of von Willebrand factor (VWF) have been reported in metastatic breast cancer compared to non-metastatic disease, suggesting a clinical link with tumor progression and dissemination [53]. While clinical prognostic validation in large patient series remains limited for other EV-associated proteins such as MYH9, ANGPTL6, CHMP1A, and VTA1, these proteins have been implicated in key processes relevant to tumor biology and warrant further investigation in well-characterized patient cohorts.

Few limitations should be acknowledged when interpreting these findings. First, the meta-analysis combined EV proteomes derived from cell lines and patient biofluids, which differ substantially in biological context and background protein composition. Second, plasma, serum, and urine datasets were pooled without stratified meta-analyses because of the small number of eligible studies, and therefore matrix-specific effects may persist. Third, the included studies did not apply uniform EV characterization procedures, with variability in markers, imaging, and particle measurements that could influence the comparability of reported proteomes. Fourth, differences in proteomic platforms, labeling strategies, and isolation workflows may introduce batch or platform-specific effects that cannot be fully accounted for with the available data. Fifth, the limited number of studies precluded meaningful subgroup analyses, such as distinguishing TNBC from luminal subtypes. Finally, selective reporting and publication bias are well-recognized challenges in proteomic literature, as many studies report only statistically significant proteins rather than full detection lists. This selective reporting may inflate apparent concordance across studies and bias vote-counting and combined *p*-value approaches. Together, these factors indicate that the results should be interpreted cautiously until validated in larger, methodologically standardized cohorts.

## 5. Conclusions

This systematic review highlights that extracellular vesicles (EVs) released from breast cancer cells display distinctive proteomic patterns enriched in pathways governing adhesion, signaling, immune modulation, and tumor progression. Among the consistently identified proteins, integrin β1 (ITGB1), myosin heavy chain (MYH9), von Willebrand factor (VWF), and vesicle-trafficking regulators such as VTA1, CHMP1A, and CLTA exemplify the dual role of breast cancer–derived EVs. These vesicles not only transport bioactive molecules that drive angiogenesis, invasion, and metastasis but also contain structural components that enhance vesicle formation and intercellular communication within the tumor microenvironment. Collectively, the evidence supports EV proteomics as a promising, minimally invasive strategy for discovering biomarkers that could refine breast cancer diagnosis, prognosis, and therapeutic monitoring. However, the limited number of available studies and methodological heterogeneity emphasize the need for larger, standardized investigations to validate these findings and clarify the biological functions of key EV-associated proteins. Future efforts that integrate proteomic signatures with clinical and molecular data may advance the use of EV-based biomarkers in precision oncology, ultimately improving early detection and personalized management of breast cancer.

## Figures and Tables

**Table 1:** Top 10 enriched pathways in breast cancer EVs. Pathway enrichment analysis of 628 significantly differentially expressed proteins identified in extracellular vesicles (EVs) from breast cancer samples were performed using FunRich. Bonferroni, BH-adjusted *p*-values, and Storey–Tibshirani q-values are reported. Pathways with q < 0.05 are indicated as statistically significant following multiple-testing correction.

Altered Pathway	Number of Proteins from Dataset	Proteins from Background Dataset	*p*-Value	Bonferroni Method	BH Method	Q-Value (Storey–Tibshirani Method)	Altered Proteins from the Dataset
Integrin family cell surface interactions	96	1375	1.42 × 10^−8^	2.37021 × 10^−5^	2.15474 × 10^−8^	9.5009 × 10^−6^	CLTA; CEBPZ; AHSG; COL1A1; FGG; FGA; FGB; ITGA6; FBN1; TLN1; TYK2; GSK3B; MDM4; GAB1; DMP1; TIAM1; ITGAE; ACTN1; MED1; ALDH9A1; ARPC1B; ARPC2; ARPC3; BAIAP2; CALM1; CALM2; CALM3; CDC42; CDH1; CLIP1; COL1A2; COL3A1; COL5A1; COL7A1; CSF1R; CSK; CTNNB1; F11R; FN1; FYN; GNA13; GNAI1; GNAO1; HSPA1A; HSPA1B; ICAM1; ICAM2; ITGA2B; ITGB1; ITGB3; LAMA2; LIMA1; MMP2; NCKAP1; NDRG1; NFKB2; PECAM1; PGK1; PPP2R1A; PPP5C; PRKCD; PTK2; PTPRC; PTPRJ; PXN; RAP1A; RAP1B; RUNX1; STAT5A; STAT5B; TNC; VCAM1; YES1; ZYX; LAMA3; INS; C3; CLU; GSN; FTH1; F10; KNG1; MST1; LRP1; VTN; PGM1; NF1; AP2A1; ARF1; CAPN2; CTTN; DNM1; DNM2; MMP3; RAB11A; SPAG9;
Beta1 integrin cell surface interactions	90	1348	4.67 × 10^−7^	0.000779588	4.1031 × 10^−5^	0.000180918	CLTA; CEBPZ; AHSG; COL1A1; FGG; FGA; FGB; ITGA6; FBN1; TLN1; TYK2; GSK3B; MDM4; GAB1; DMP1; TIAM1; ITGAE; ACTN1; MED1; ALDH9A1; ARPC1B; ARPC2; ARPC3; BAIAP2; CALM1; CALM2; CALM3; CDC42; CDH1; CLIP1; COL1A2; COL3A1; COL5A1; COL7A1; CSF1R; CSK; CTNNB1; FN1; FYN; GNA13; GNAI1; GNAO1; HSPA1A; HSPA1B; ICAM1; ITGA2B; ITGB1; ITGB3; LAMA2; LIMA1; MMP2; NCKAP1; NDRG1; NFKB2; PGK1; PPP2R1A; PPP5C; PRKCD; PTK2; PTPRC; PTPRJ; PXN; RAP1A; RAP1B; RUNX1; STAT5A; STAT5B; TNC; VCAM1; YES1; ZYX; LAMA3; INS; CLU; GSN; FTH1; MST1; LRP1; VTN; PGM1; NF1; AP2A1; ARF1; CAPN2; CTTN; DNM1; DNM2; MMP3; RAB11A; SPAG9;
Proteoglycan syndecan-mediated signaling events	89	1342	7.69 × 10^−7^	0.001281882	6.40941 × 10^−5^	0.000282611	CLTA; CEBPZ; F2; AHSG; COL1A1; FGG; FGA; FGB; ITGA6; TLN1; TYK2; GSK3B; MDM4; GAB1; DMP1; TIAM1; ITGAE; ACTN1; MED1; ALDH9A1; ARPC1B; ARPC2; ARPC3; BAIAP2; CALM1; CALM2; CALM3; CDC42; CDH1; CLIP1; COL1A2; CSF1R; CSK; CTNNB1; EZR; FN1; FYN; GNA13; GNAI1; GNAO1; HSPA1A; HSPA1B; ICAM1; ITGA2B; ITGB1; ITGB3; LIMA1; MMP2; NCKAP1; NDRG1; NFKB2; PGK1; PPP2R1A; PPP5C; PRKCD; PTK2; PTPRC; PTPRJ; PXN; RAP1A; RAP1B; RUNX1; STAT5A; STAT5B; TNC; YES1; ZYX; LAMA3; INS; CLU; BSG; GSN; FTH1; KNG1; MST1; LRP1; VTN; PGM1; NF1; AP2A1; ARF1; CAPN2; CTTN; DNM1; DNM2; MMP3; RAB11A; SDCBP; SPAG9;
TRAIL signaling pathway	86	1325	3.37 × 10^−6^	0.005617398	0.000170224	0.000750571	CLTA; CEBPZ; AHSG; COL1A1; FGG; FGA; FGB; ITGA6; TLN1; TYK2; GSK3B; MDM4; NUMA1; GAB1; DMP1; TIAM1; ITGAE; ACTN1; MED1; ALDH9A1; ARPC1B; ARPC2; ARPC3; BAIAP2; CALM1; CALM2; CALM3; CDC42; CDH1; CFL2; CLIP1; COL1A2; CSF1R; CSK; CTNNB1; FN1; FYN; GNA13; GNAI1; GNAO1; HSPA1A; HSPA1B; ICAM1; ITGA2B; ITGB1; ITGB3; LIMA1; MMP2; NCKAP1; NDRG1; NFKB2; PGK1; PPP2R1A; PPP5C; PRKCD; PTK2; PTPRC; PTPRJ; PXN; RAP1A; RAP1B; RUNX1; STAT5A; STAT5B; TFAP2A; YES1; ZYX; LAMA3; INS; CLU; GSN; FTH1; MST1; LRP1; VTN; PGM1; NF1; AP2A1; ARF1; CAPN2; CTTN; DNM1; DNM2; MMP3; RAB11A; SPAG9;
Syndecan-1-mediated signaling events	85	1297	2.64 × 10^−6^	0.004401549	0.000151778	0.000669234	CLTA; CEBPZ; AHSG; COL1A1; FGG; FGA; FGB; ITGA6; TLN1; TYK2; GSK3B; MDM4; GAB1; DMP1; TIAM1; ITGAE; ACTN1; MED1; ALDH9A1; ARPC1B; ARPC2; ARPC3; BAIAP2; CALM1; CALM2; CALM3; CDC42; CDH1; CLIP1; COL1A2; CSF1R; CSK; CTNNB1; FN1; FYN; GNA13; GNAI1; GNAO1; HSPA1A; HSPA1B; ICAM1; ITGA2B; ITGB1; ITGB3; LIMA1; MMP2; NCKAP1; NDRG1; NFKB2; PGK1; PPP2R1A; PPP5C; PRKCD; PTK2; PTPRC; PTPRJ; PXN; RAP1A; RAP1B; RUNX1; STAT5A; STAT5B; YES1; ZYX; LAMA3; INS; CLU; BSG; GSN; FTH1; MST1; LRP1; VTN; PGM1; NF1; AP2A1; ARF1; CAPN2; CTTN; DNM1; DNM2; MMP3; RAB11A; SDCBP; SPAG9;
Glypican pathway	85	1335	8.83 × 10^−6^	0.014723522	0.000226516	0.000998777	CLTA; CEBPZ; AHSG; COL1A1; FGG; FGA; FGB; ITGA6; TLN1; TYK2; GSK3B; MDM4; GAB1; DMP1; TIAM1; ITGAE; ACTN1; MED1; ALDH9A1; ARPC1B; ARPC2; ARPC3; BAIAP2; CALM1; CALM2; CALM3; CDC42; CDH1; CLIP1; COL1A2; CSF1R; CSK; CTNNB1; FN1; FYN; GNA13; GNAI1; GNAO1; HSPA1A; HSPA1B; ICAM1; ITGA2B; ITGB1; ITGB3; LIMA1; MMP2; NCKAP1; NDRG1; NFKB2; PGK1; PPP2R1A; PPP5C; PRKCD; PTK2; PTPRC; PTPRJ; PXN; RAP1A; RAP1B; RUNX1; STAT5A; STAT5B; YES1; ZYX; LAMA3; INS; CLU; SERPINC1; GSN; FTH1; MST1; LRP1; VTN; SHH; PGM1; NF1; AP2A1; ARF1; CAPN2; CTTN; DNM1; DNM2; MMP3; RAB11A; SPAG9;
PAR1-mediated thrombin signaling events	85	1296	2.55 × 10^−6^	0.004258985	0.000151778	0.000669234	CLTA; CEBPZ; F2; AHSG; COL1A1; FGG; FGA; FGB; ITGA6; TLN1; TYK2; GSK3B; MDM4; GAB1; DMP1; TIAM1; ITGAE; ACTN1; MED1; ALDH9A1; ARPC1B; ARPC2; ARPC3; BAIAP2; CALM1; CALM2; CALM3; CDC42; CDH1; CLIP1; COL1A2; CSF1R; CSK; CTNNB1; FN1; FYN; GNA13; GNAI1; GNAO1; HSPA1A; HSPA1B; ICAM1; ITGA2B; ITGB1; ITGB3; LIMA1; MMP2; NCKAP1; NDRG1; NFKB2; PGK1; PLCB3; PPP2R1A; PPP5C; PRKCD; PTK2; PTPRC; PTPRJ; PXN; RAP1A; RAP1B; RUNX1; STAT5A; STAT5B; YES1; ZYX; LAMA3; INS; CLU; GSN; FTH1; MST1; LRP1; VTN; PGM1; NF1; AP2A1; ARF1; CAPN2; CTTN; DNM1; DNM2; MMP3; RAB11A; SPAG9;
Thrombin/protease-activated receptor (PAR) pathway	85	1297	2.64 × 10^−6^	0.004401549	0.000151778	0.000669234	CLTA; CEBPZ; F2; AHSG; COL1A1; FGG; FGA; FGB; ITGA6; TLN1; TYK2; GSK3B; MDM4; GAB1; DMP1; TIAM1; ITGAE; ACTN1; MED1; ALDH9A1; ARPC1B; ARPC2; ARPC3; BAIAP2; CALM1; CALM2; CALM3; CDC42; CDH1; CLIP1; COL1A2; CSF1R; CSK; CTNNB1; FN1; FYN; GNA13; GNAI1; GNAO1; HSPA1A; HSPA1B; ICAM1; ITGA2B; ITGB1; ITGB3; LIMA1; MMP2; NCKAP1; NDRG1; NFKB2; PGK1; PLCB3; PPP2R1A; PPP5C; PRKCD; PTK2; PTPRC; PTPRJ; PXN; RAP1A; RAP1B; RUNX1; STAT5A; STAT5B; YES1; ZYX; LAMA3; INS; CLU; GSN; FTH1; MST1; LRP1; VTN; PGM1; NF1; AP2A1; ARF1; CAPN2; CTTN; DNM1; DNM2; MMP3; RAB11A; SPAG9;
Alpha9 beta1 integrin signaling events	85	1302	3.11 × 10^−6^	0.005184572	0.000167244	0.000737431	CLTA; CEBPZ; AHSG; COL1A1; FGG; FGA; FGB; ITGA6; TLN1; TYK2; GSK3B; MDM4; GAB1; DMP1; TIAM1; ITGAE; ACTN1; MED1; ALDH9A1; ARPC1B; ARPC2; ARPC3; BAIAP2; CALM1; CALM2; CALM3; CDC42; CDH1; CLIP1; COL1A2; CSF1R; CSK; CTNNB1; FN1; FYN; GNA13; GNAI1; GNAO1; HSPA1A; HSPA1B; ICAM1; ITGA2B; ITGB1; ITGB3; LIMA1; MMP2; NCKAP1; NDRG1; NFKB2; PGK1; PPP2R1A; PPP5C; PRKCD; PTK2; PTPRC; PTPRJ; PXN; RAP1A; RAP1B; RUNX1; STAT5A; STAT5B; TNC; VCAM1; YES1; ZYX; LAMA3; INS; CLU; GSN; FTH1; MST1; LRP1; VTN; PGM1; NF1; AP2A1; ARF1; CAPN2; CTTN; DNM1; DNM2; MMP3; RAB11A; SPAG9;
Endothelins	85	1304	3.32 × 10^−6^	0.005533176	0.000170224	0.000750571	CLTA; CEBPZ; AHSG; COL1A1; FGG; FGA; FGB; ITGA6; TLN1; TYK2; GSK3B; MDM4; GAB1; DMP1; TIAM1; ITGAE; ACTN1; MED1; ALDH9A1; ARPC1B; ARPC2; ARPC3; BAIAP2; CALM1; CALM2; CALM3; CDC42; CDH1; CLIP1; COL1A2; COL3A1; CSF1R; CSK; CTNNB1; FN1; FYN; GNA13; GNAI1; GNAO1; HSPA1A; HSPA1B; ICAM1; ITGA2B; ITGB1; ITGB3; LIMA1; MMP2; NCKAP1; NDRG1; NFKB2; PGK1; PLCB3; PPP2R1A; PPP5C; PRKCD; PTK2; PTPRC; PTPRJ; PXN; RAP1A; RAP1B; RUNX1; STAT5A; STAT5B; YES1; ZYX; LAMA3; INS; CLU; GSN; FTH1; MST1; LRP1; VTN; PGM1; NF1; AP2A1; ARF1; CAPN2; CTTN; DNM1; DNM2; MMP3; RAB11A; SPAG9;

## Data Availability

All data supporting the findings of this study are available within the manuscript and its Supplementary Information Files.

## Supplementary Materials

The following supporting information can be downloaded at: https://www.mdpi.com/article/10.3390/cells15030231/s1.

## References

[1] Sung H., Ferlay J., Siegel R.L., Laversanne M., Soerjomataram I., Jemal A., Bray F. (2021). Global Cancer Statistics 2020: GLOBOCAN Estimates of Incidence and Mortality Worldwide for 36 Cancers in 185 Countries. CA A Cancer J. Clin..

[2] Wang L. (2017). Early Diagnosis of Breast Cancer. Sensors.

[3] Gilbert F.J., Pinker-Domenig K., Hodler J., Kubik-Huch R.A., von Schulthess G.K. (2019). Diagnosis and Staging of Breast Cancer: When and How to Use Mammography, Tomosynthesis, Ultrasound, Contrast-Enhanced Mammography, and Magnetic Resonance Imaging. Diseases of the Chest, Breast, Heart and Vessels 2019–2022: Diagnostic and Interventional Imaging.

[4] Tsarouchi M., Hoxhaj A., Portaluri A., Sung J., Sechopoulos I., Pinker-Domenig K., Mann R.M. (2025). Breast cancer staging with contrast-enhanced imaging. The benefits and drawbacks of MRI, CEM, and dedicated breast CT. Eur. J. Radiol..

[5] Panagopoulou M., Esteller M., Chatzaki E. (2021). Circulating Cell-Free DNA in Breast Cancer: Searching for Hidden Information towards Precision Medicine. Cancers.

[6] Beca F., Polyak K. (2016). Intratumor Heterogeneity in Breast Cancer. Adv. Exp. Med. Biol..

[7] Alimirzaie S., Bagherzadeh M., Akbari M.R. (2019). Liquid Biopsy in Breast Cancer: A Comprehensive Review. Clin. Genet..

[8] Wang H., Zhang Y., Zhang H., Cao H., Mao J., Chen X., Wang L., Zhang N., Luo P., Xue J. (2024). Liquid biopsy for human cancer: Cancer screening, monitoring, and treatment. Medcomm.

[9] Chen Y., Xue F., Russo A., Wan Y. (2021). Proteomic Analysis of Extracellular Vesicles Derived from MDA-MB-231 Cells in Microgravity. Protein J..

[10] Rontogianni S., Synadaki E., Li B., Liefaard M.C., Lips E.H., Wesseling J., Wu W., Altelaar M. (2019). Proteomic profiling of extracellular vesicles allows for human breast cancer subtyping. Commun. Biol..

[11] Pan W., Feng J., Luo T., Tan Y., Situ B., Nieuwland R., Guo J., Liu C., Zhang H., Chen J. (2022). Rapid and efficient isolation platform for plasma extracellular vesicles: EV-FISHER. J. Extracell. Vesicles.

[12] Van Niel G., D’Angelo G., Raposo G. (2018). Shedding light on the cell biology of extracellular vesicles. Nat. Rev. Mol. Cell Biol..

[13] Sódar B.W., Kittel Á., Pálóczi K., Vukman K.V., Osteikoetxea X., Szabó-Taylor K., Németh A., Sperlágh B., Baranyai T., Giricz Z. (2016). Low-density lipoprotein mimics blood plasma-derived exosomes and microvesicles during isolation and detection. Sci. Rep..

[14] Willms E., Johansson H.J., Mäger I., Lee Y., Blomberg K.E.M., Sadik M., Alaarg A., Smith C.E., Lehtiö J., EL Andaloussi S. (2016). Cells release subpopulations of exosomes with distinct molecular and biological properties. Sci. Rep..

[15] Linares R., Tan S., Gounou C., Arraud N., Brisson A.R. (2015). High-speed centrifugation induces aggregation of extracellular vesicles. J. Extracell. Vesicles.

[16] Tóth E.Á., Turiák L., Visnovitz T., Cserép C., Mázló A., Sódar B.W., Försönits A.I., Petővári G., Sebestyén A., Komlósi Z. (2021). Formation of a protein corona on the surface of extracellular vesicles in blood plasma. J. Extracell. Vesicles.

[17] Page M.J., McKenzie J.E., Bossuyt P.M., Boutron I., Hoffmann T.C., Mulrow C.D., Shamseer L., Tetzlaff J.M., Akl E.A., Brennan S.E. (2021). The PRISMA 2020 statement: An updated guideline for reporting systematic reviews. BMJ.

[18] Ahmed K.K.M., Al Dhubaib B.E. (2011). Zotero: A bibliographic assistant to researcher. J. Pharmacol. Pharmacother..

[19] Llambrich M., Correig E., Gumà J., Brezmes J., Cumeras R. (2021). Amanida: An R package for meta-analysis of metabolomics non-integral data. Bioinformatics.

[20] Pathan M., Keerthikumar S., Ang C.-S., Gangoda L., Quek C.Y., Williamson N.A., Mouradov D., Sieber O.M., Simpson R.J., Salim A. (2015). FunRich: An open access standalone functional enrichment and interaction network analysis tool. Proteomics.

[21] Szklarczyk D., Kirsch R., Koutrouli M., Nastou K., Mehryary F., Hachilif R., Gable A.L., Fang T., Doncheva N.T., Pyysalo S. (2022). The STRING database in 2023: Protein–protein association networks and functional enrichment analyses for any sequenced genome of interest. Nucleic Acids Res..

[22] Kim H.I., Park J., Zhu Y., Wang X., Han Y., Zhang D. (2024). Recent advances in extracellular vesicles for therapeutic cargo delivery. Exp. Mol. Med..

[23] Maas S.L.N., Breakefield X.O., Weaver A.M. (2017). Extracellular Vesicles: Unique Intercellular Delivery Vehicles. Trends Cell Biol..

[24] Hessvik N.P., Llorente A. (2018). Current knowledge on exosome biogenesis and release. Cell. Mol. Life Sci..

[25] Kajimoto T., Okada T., Miya S., Zhang L., Nakamura S.-I. (2013). Ongoing activation of sphingosine 1-phosphate receptors mediates maturation of exosomal multivesicular endosomes. Nat. Commun..

[26] Trajkovic K., Hsu C., Chiantia S., Rajendran L., Wenzel D., Wieland F., Schwille P., Brügger B., Simons M. (2008). Ceramide Triggers Budding of Exosome Vesicles into Multivesicular Endosomes. Science.

[27] Cvjetkovic A., Jang S.C., Konečná B., Höög J.L., Sihlbom C., Lässer C., Lötvall J. (2016). Detailed Analysis of Protein Topology of Extracellular Vesicles–Evidence of Unconventional Membrane Protein Orientation. Sci. Rep..

[28] Jin Y., Ma L., Zhang W., Yang W., Feng Q., Wang H. (2022). Extracellular signals regulate the biogenesis of extracellular vesicles. Biol. Res..

[29] Ma F., Vayalil J., Lee G., Wang Y., Peng G. (2021). Emerging role of tumor-derived extracellular vesicles in T cell suppression and dysfunction in the tumor microenvironment. J. Immunother. Cancer.

[30] Santoro J., Carrese B., Peluso M.S., Coppola L., D’aiuto M., Mossetti G., Salvatore M., Smaldone G. (2023). Influence of Breast Cancer Extracellular Vesicles on Immune Cell Activation: A Pilot Study. Biology.

[31] Ozawa P.M.M., Alkhilaiwi F., Cavalli I.J., Malheiros D., de Souza Fonseca Ribeiro E.M., Cavalli L.R. (2018). Extracellular vesicles from triple-negative breast cancer cells promote proliferation and drug resistance in non-tumorigenic breast cells. Breast Cancer Res. Treat..

[32] Leone I., Santoro J., Soricelli A., Febbraro A., Santoriello A., Carrese B. (2024). Triple-Negative Breast Cancer EVs Modulate Growth and Migration of Normal Epithelial Lung Cells. Int. J. Mol. Sci..

[33] Fuentes P., Sesé M., Guijarro P.J., Emperador M., Sánchez-Redondo S., Peinado H., Hümmer S., Cajal S.R.Y. (2020). ITGB3-mediated uptake of small extracellular vesicles facilitates intercellular communication in breast cancer cells. Nat. Commun..

[34] Huang X., Lai S., Qu F., Li Z., Fu X., Li Q., Zhong X., Wang C., Li H. (2022). CCL18 promotes breast cancer progression by exosomal miR-760 activation of ARF6/Src/PI3K/Akt pathway. Mol. Ther.—Oncol..

[35] Li B., Lu Y., Yu L., Han X., Wang H., Mao J., Shen J., Wang B., Tang J., Li C. (2017). miR-221/222 promote cancer stem-like cell properties and tumor growth of breast cancer via targeting PTEN and sustained Akt/NF-κB/COX-2 activation. Chem. Interact..

[36] Hurwitz S.N., Meckes D.G. (2019). Extracellular Vesicle Integrins Distinguish Unique Cancers. Proteomes.

[37] Hynes R.O. (1992). Integrins: Versatility, modulation, and signaling in cell adhesion. Cell.

[38] Zhang W., Li Y., Chen G., Yang X., Hu J., Zhang X., Feng G., Wang H. (2022). Integrin α6-Targeted Molecular Imaging of Central Nervous System Leukemia in Mice. Front. Bioeng. Biotechnol..

[39] Zhou Z., Qu J., He L., Peng H., Chen P., Zhou Y. (2018). α6-Integrin alternative splicing: Distinct cytoplasmic variants in stem cell fate specification and niche interaction. Stem Cell Res. Ther..

[40] Gang E.J., Na Kim H., Hsieh Y.-T., Ruan Y., Ogana H.A., Lee S., Pham J., Geng H., Park E., Klemm L. (2020). Integrin α6 mediates the drug resistance of acute lymphoblastic B-cell leukemia. Blood.

[41] McDonald P.C., Dedhar S. (2022). New Perspectives on the Role of Integrin-Linked Kinase (ILK) Signaling in Cancer Metastasis. Cancers.

[42] Asada T., Nakahata S., Fauzi Y.R., Ichikawa T., Inoue K., Shibata N., Fujii Y., Imamura N., Hiyoshi M., Nanashima A. (2022). Integrin α6A (ITGA6A)-type Splice Variant in Extracellular Vesicles Has a Potential as a Novel Marker of the Early Recurrence of Pancreatic Cancer. Anticancer Res..

[43] Arraud N., Linares R., Tan S., Gounou C., Pasquet J., Mornet S., Brisson A.R. (2014). Extracellular vesicles from blood plasma: Determination of their morphology, size, phenotype and concentration. J. Thromb. Haemost..

[44] Gyorgy B., Modos K., Pallinger E., Paloczi K., Pasztoi M., Misjak P., Deli M.A., Sipos A., Szalai A., Voszka I. (2011). Detection and Isolation of Cell-Derived Microparticles Are Compromised by Protein Complexes Resulting from Shared Biophysical Parameters. Blood.

[45] Simonsen J.B. (2017). What Are We Looking At? Extracellular Vesicles, Lipoproteins, or Both?. Circ. Res..

[46] héry C., Witwer K.W., Aikawa E., Alcaraz M.J., Anderson J.D., Andriantsitohaina R., Antoniou A., Arab T., Archer F., Atkin-Smith G.K. (2018). Minimal information for studies of extracellular vesicles 2018 (MISEV2018): A position statement of the International Society for Extracellular Vesicles and update of the MISEV2014 guidelines. J. Extracell. Vesicles.

[47] Böing A.N., van der Pol E., Grootemaat A.E., Coumans F.A.W., Sturk A., Nieuwland R. (2014). Single-step isolation of extracellular vesicles by size-exclusion chromatography. J. Extracell. Vesicles.

[48] Andreu Z., Hidalgo M.R., Masiá E., Romera-Giner S., Malmierca-Merlo P., López-Guerrero J.A., García-García F., Vicent M.J. (2024). Comparative profiling of whole-cell and exosome samples reveals protein signatures that stratify breast cancer subtypes. Cell. Mol. Life Sci..

[49] Xu G., Huang R., Wumaier R., Lyu J., Huang M., Zhang Y., Chen Q., Liu W., Tao M., Li J. (2024). Proteomic Profiling of Serum Extracellular Vesicles Identifies Diagnostic Signatures and Therapeutic Targets in Breast Cancer. Cancer Res..

[50] Yang H., Zhang M., Mao X.-Y., Chang H., Perez-Losada J., Mao J.-H. (2021). Distinct Clinical Impact and Biological Function of Angiopoietin and Angiopoietin-like Proteins in Human Breast Cancer. Cells.

[51] Colombo M., Raposo G., Théry C. (2014). Biogenesis, secretion, and intercellular interactions of exosomes and other extracellular vesicles. Annu. Rev. Cell Dev. Biol..

[52] Sun Q., Zhou C., Ma R., Guo Q., Huang H., Hao J., Liu H., Shi R., Liu B. (2018). Prognostic value of increased integrin-beta 1 expression in solid cancers: A meta-analysis. OncoTargets Ther..

[53] Goh C.Y., Patmore S., Smolenski A., Howard J., Evans S., O’SUllivan J., McCann A. (2021). The role of von Willebrand factor in breast cancer metastasis. Transl. Oncol..

