# Supplementary material for: Extracellular Vesicle Associated Proteomic Biomarkers in Breast Cancer: A Systematic Review and Meta-Analysis
Source: Cells. 2026 Jan 26;15(3):231. doi: 10.3390/cells15030231 (PMC12896969; doi:10.3390/cells15030231)

Supplementary Figure S1: Gene ontology enrichment analysis of differentially expressed proteins in extracellular vesicles (EVs) from breast cancer patients. The bar chart illustrates the percentage of genes associated with each biological process (blue bars), alongside the statistical significance of enrichment (-log10 p-value, red bars).

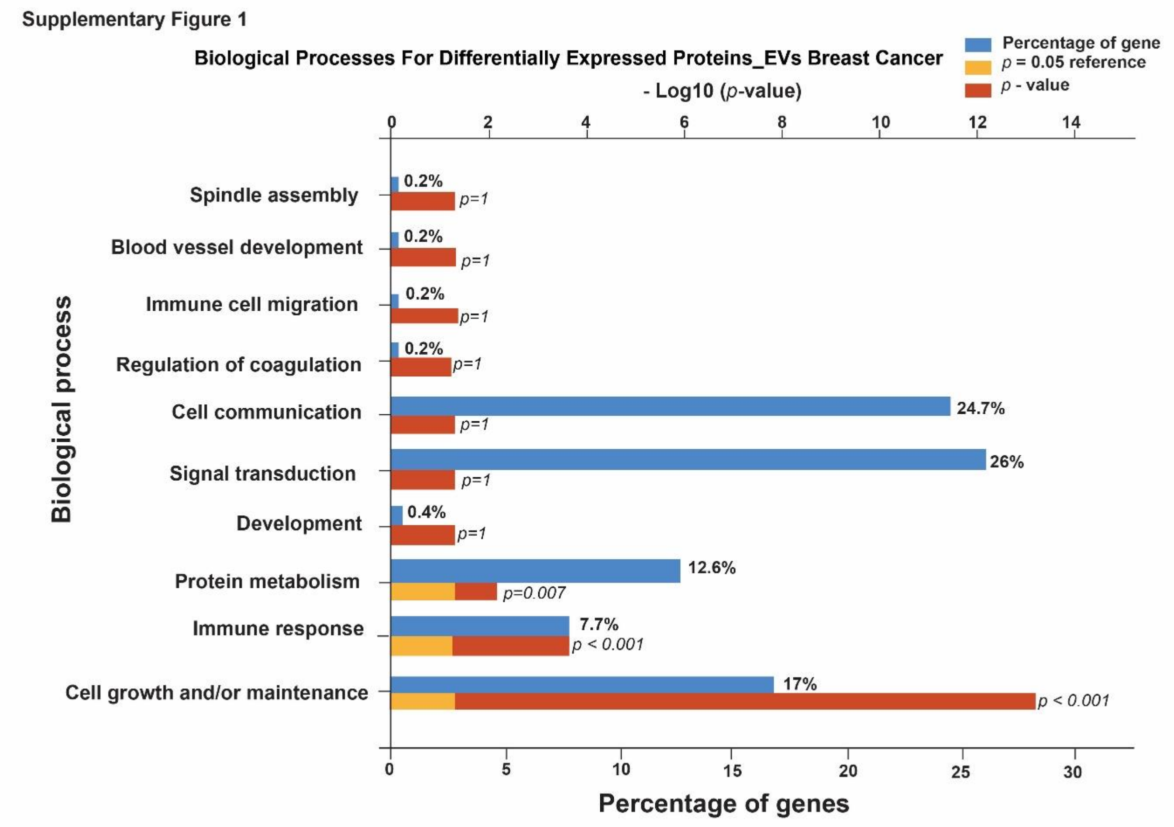

Supplement: Supplementary file 1 [file cells-15-00231-s001.zip › Supplementary Figure S1.pdf]
