# Supplementary material for: Extracellular Vesicle Associated Proteomic Biomarkers in Breast Cancer: A Systematic Review and Meta-Analysis
Source: Cells. 2026 Jan 26;15(3):231. doi: 10.3390/cells15030231 (PMC12896969; doi:10.3390/cells15030231)
Supplement: Supplementary file 1 [file cells-15-00231-s001.zip › Supplementary Methods – Section S1.pdf]

Supplementary Methods – Section S1: Full Search Strings

NCBI (PubMed):
("extracellular vesicles"[Title/Abstract] OR "exosomes"[Title/Abstract] OR "microvesicles"[Title/Abstract]) AND ("breast cancer"[Title/Abstract]) AND ("proteomics"[Title/Abstract] OR "protein profiling"[Title/Abstract])

Medline (EBSCO):
(extracellular vesicles OR exosomes OR microvesicles) AND (breast cancer) AND (proteomics OR "mass spectrometry" OR "protein expression")

Academic Search Ultimate:
TX (extracellular vesicles OR exosomes OR microvesicles) AND TX (breast cancer) AND TX (proteomics OR "protein biomarkers")

CINAHL Plus:
(MH "Extracellular Vesicles" OR exosomes OR microvesicles) AND (MH "Breast Neoplasms" OR "breast cancer") AND (proteomics OR "protein analysis")
